# The association of mobility disability and obesity with risk of unemployment in two cohorts from Sweden

**DOI:** 10.1186/s12889-019-6627-2

**Published:** 2019-03-28

**Authors:** Mattias Norrbäck, Per Tynelius, Gerd Ahlström, Finn Rasmussen

**Affiliations:** 1 Clinical Epidemiological Group, Department of Medicine, Karolinska Institute, Stockholm, Sweden; 2 Centre of Epidemiology and Community Medicine, Stockholm County Council, Health Care Services, Stockholm, Sweden; 3 Department of Health Sciences, Faculty of Medicine, Lund University, Box 157, 221 00 Lund, Sweden

**Keywords:** Disability, Weight, Cohort study, Work participation, Unemployment

## Abstract

**Background:**

People with mobility disability (MD) or obesity often have more health problems and are less able to participate in work than individuals without these conditions. This study investigated whether people burdened with MD *and* obesity have a greater risk of unemployment than people with either one (MD only or obesity only) or none of these conditions.

**Methods:**

The study included two Swedish population-based cohorts, a national cohort (*n* = 39,947) and a regional cohort (*n* = 40,088). Six exposure groups were created using baseline self-reported data on MD and body mass index from participants aged 19 to 64 years. The MD definition differed between the cohorts. Various sources of socio-demographic factors were used to address confounding. Participants’ risks of unemployment were assessed longitudinally in a nationwide register with objective data and with almost no loss of follow-up (< 1%). Cox regression was used to analyse associations of MD and/or obesity (BMI ≥ 30) with risk of any (≥1 day) and long-term unemployment (≥90 days during two consecutive years). Quantile regression was used to estimate participants’ unemployment risks as average days of unemployment. Normal-weight people without MD were used as a reference group. The Wald test was applied for specific group comparisons other than to the reference group.

**Results:**

In summary, the groups with MD and the obese group without MD had a higher risk of becoming unemployed than the reference group (regional survey adjusted hazard ratio range: 1.30–1.59; 95% CI range: 1.06–1.90, national survey adjusted hazard ratio range: 1.11–1.34; 95% CI range: 0.88–1.81). The obese group with MD did not differ from the groups with MD only or obesity only in terms of unemployment risk.

**Conclusions:**

People with MD and/or obesity are vulnerable groups at risk of prolonged unemployment during their working life in a country with a highly developed welfare system.

**Electronic supplementary material:**

The online version of this article (10.1186/s12889-019-6627-2) contains supplementary material, which is available to authorized users.

## Background

Both mobility disability (MD) and obesity are common conditions worldwide [1, 2] that largely contribute to the global disease burden, making them major public health challenges in modern times [3, 4]. In Sweden, approximately 12% and 14% of women and men, respectively, are obese defined as having a body mass index (BMI) greater than or equal to 30 based on self-reported data of weight and height [5]. MD is a broad concept with no agreed upon definition. The International Classification of Functioning, Disability and Health (ICF) [6] framework seeks to define and understand the emergence of disabilities. According to the ICF framework, MD may arise from accidents, injuries, or chronic diseases causing the restriction of bodily functions, such as, diseases in the musculoskeletal system, for example, rheumatoid arthritis, osteoarthritis, and disorders related to the spinal system. Further, MD is also common in people with musculoskeletal pain and other related chronic health problems [7, 8]. In population-based surveys, respondents are often defined as having MD when they have disagreed to multiple (or single) statements about mobility, for instance; “*I am able to walk a short distance of 100 m”* and/or “*I am able to climb a set of stairs without problems”*. Based on such definitions, approximately 7% of Swedish adults are living with MD [9], and well over 20% in the US adult population [10].

MD and obesity are more likely to co-exist over the life course. A Swedish population-based study found that compared with normal weight people without MD, people with obesity (middle-aged women especially), were almost four times as likely to develop MD after 8 years of follow-up [11]. The study also found that people with MD were more likely to increase in BMI over the study period [11]. Previous studies have reported that around 0.5–1% of Swedish adults live with both MD and obesity [11–13]. This is a relatively large and overlooked group of people that has an excess risk of poor health, reduced functioning, and decreased quality of life beyond the risk attributed to MD or obesity alone [14, 15]. Further, living with both MD *and* obesity may reduce peoples work ability over the life course. Reduced work ability is associated with frequent and longer sick leave episodes [16, 17], and has been associated with subsequent unemployment and disability pension for people of the general work force, especially in women and older employees [18].

We have previously shown that people with MD and obesity are more likely to experience job stress [19] and with greater risk of disability pension [13] than people without these conditions. Less is known about whether, and if so to what extent, the co-existence of MD *and* obesity are associated with the possibility to retain employment. The primary objective of this study was; therefore, to investigate whether people with MD *and* obesity are at increased risk of unemployment compared to people having one (MD only or obesity only) or none of these conditions (normal weight without MD). Moreover, it is possible that unemployment risk can vary in people with MD, depending on the MD definition used. As a secondary objective, we therefore examined the robustness of associations in two different large population-based cohorts established by different definitions of MD (as described below).

## Methods

### Design, study population, and data

This is a prospective cohort study based on population-based surveys and one national register with up to 16 years of follow-up. Samples from two separate populations including people of working age (ages 19–64) were included. The first study population consisted of participants who took part in the National Survey of Living Conditions (ULF/SILC) between 1996 and 2011 (baseline). This survey is conducted annually by Statistics Sweden on random nationwide samples of the Swedish population [20]. The second study population was based on pooled data comprising individuals from two of the Stockholm Public Health Surveys (SPHS) conducted in 2002 and 2006 (baseline). SPHS are based on stratified random samples from the total population of Stockholm (capital of Sweden) County (*n* = 1,900,000), aged 18–84 years [21]. The two study populations were followed up in the Longitudinal Integration Database for Health Insurance and Labour Market Studies (LISA) between 1997 and 2012 in terms of unemployment (outcome). LISA is a national database administered by Statistics Sweden and contains annually updated labour market data of all individuals 16 years of age or older who are registered in Sweden [22]. Further, information on disability benefits (part-time or full-time) was collected from the Swedish Social Insurance Register between 1990 and 2012. Information on mortality and emigration was taken from the Cause of Death Register and the Immigration Register, held by Statistics Sweden.

### Exposure

Information on mobility status differed between the ULF/SILC and SPHS cohorts. In ULF/SILC, people were categorized as having MD by answering “no” on question i) AND “no” on EITHER question ii) OR “no” on question iii):

i. “Can you run a short distance, approximately 100 m, if you are in a hurry?”
ii. “Can you go on and off a bus without experiencing any problems?”
iii. “Can you take a short walk for about five minutes at a moderately high pace?”

In the SPHS cohort, mobility status was evaluated by a question originating from the EuroQol EQ-5D-3 L self-rating scale [23]. Here, people who had answered “yes” on one of the following two alternatives, “I have some problems in walking about” OR “I am confined to bed”, were categorized as having MD. Self-reported height and weight were used to calculate body mass index (BMI) (kg/m^2^) in both cohorts according to WHO classification [24]. The weight categories used were normal weight (BMI 18.5–24.9), overweight (BMI 25–29.9), and obesity (BMI ≥ 30). Finally, six exposure groups were created at baseline (ULF/SILC: 1996 to 2011; SPHS: 2002 or 2006) based on combining MD and weight status: normal weight without MD (reference), normal weight with MD, overweight without MD, overweight with MD, obese without MD, and obese with MD.

### Study cohorts

Complete information on the exposure variables, i.e., height, weight, and MD status, was required for eligibility. People with underweight (BMI < 18.5 kg/m^2^) were not considered in the current study. Individuals with incomplete records or with implausible values of weight (≤ 40 kg or ≥ 250 kg), height (≤ 150 cm or ≥ 210 cm), or BMI (≥ 80 kg/m^2^) were excluded. To account for the healthy worker selection into work [25], individuals who before baseline had disability benefits [n(ULF/SILC) = 1963; n(SPHS) = 2385] or who had been long-term unemployed for ≥180 days [n(ULF/SILC) = 133; n(SPHS) = 657] were excluded from the study samples. The final ULF/SILC sample (Additional file 1) comprised 43,163 individuals (study cohort included 39,947 individuals) with complete information on mobility status, weight status, and the covariates used in the study. The final SPHS sample (Additional file 2) comprised 43,834 individuals (study cohort comprising 40,088 individuals).

### Outcome

In Sweden, there are two official ways to measure unemployment. The first way is by using the EU harmonized labour force surveys. The other way is by using unemployment records from the Swedish Employment Service (registered in the LISA database). In this study, the latter approach is used. Information on number of days in unemployment was retrieved from the LISA database. Three unemployment outcomes were defined: 1) time to first occurrence of any unemployment (≥1 day), 2) time to first occurrence of long-term unemployment (at least 90 accumulated days during two consecutive years), and 3) the average number unemployment days per year during the follow-up. Survey participation only happens once a year; however, it is technically possible to participate in more than one survey during the study period e.g. an individual can participate in a ULF/SILC survey 2000 and then later in 2008. Follow-up started the year an individual for the first time participated in either a ULF/SILC or SPHS survey and ended the year of any unemployment (1) or long-term unemployment (2), year of retirement (age of 65), year of first disability pension (1996–2012), year of emigration or death, or the end of the follow-up period (31 December 2012), whichever came first (yielding individual follow-up times between 1 and 16 years).

### Confounding factors

Socio-demographic factors including sex, age, country of birth, educational level, occupational status, and income levels were considered as possible confounders (see Table 1 for categorization) in the association between MD and weight status with risk of unemployment [26–32]. Further, short-term unemployment before baseline (between 1 and 180 days) was explored as a potential confounding factor, as people with disabilities more often have longer unemployment histories than people without disabilities [28], and a long history of unemployment predicts subsequent unemployment [33, 34]. However, because of difficulties in disentangling any potential causal mechanisms of short-term unemployment before baseline on the associations under investigation (see discussion) we chose to exclude it from the main results (from a model without this adjustment) but included it in an additional model (main model + short-term unemployment) for comparison purposes.

**Table 1:** Baseline characteristics and unemployment rates for the study samples by mobility and weight status groups

	SPHS cohort, *n* = 40,088				ULF/SILC cohort, *n* = 39,947			
Study groups	Normal weight without MD	Obese without MD	Normal weight with MD	Obese with MD	Normal weight without MD	Obese without MD	Normal weight with MD	Obese with MD
Participants in each group (%)^a^	22,886 (57.1)	3117 (7.8)	812 (2.0)	443 (1.1)	25,510 (63.9)	3684 (8.2)	419 (1.0)	239 (0.6)
Women	14,327 (62.6)	1558 (50.0)	525 (64.7)	254 (57.3)	14,478 (56.8)	1524 (41.4)	291 (69.5)	142 (59.4)
Age, mean (SD)	40.0 (12.2)	44.9 (11.1)	44.9 (12.2)	49.7 (10.6)	38.0 (12.4)	42.6 (11.4)	47.3 (11.9)	48.7 (10.8)
Age class, 18–29	5234 (22.9)	309 (9.9)	111 (13.7)	26 (5.9)	7924 (31.1)	541 (14.7)	39 (9.3)	14 (5.9)
30–54	13,891 (60.7)	2035 (65.3)	478 (58.9)	221 (49.9)	14,331 (56.2)	2476 (67.2)	232 (55.4)	134 (56.1)
55 and older	3761 (16.4)	773 (24.8)	223 (27.5)	196 (44.2)	3255 (12.8)	667 (18.1)	148 (35.3)	91 (38.1)
Country of Birth, Sweden	19,806 (86.5)	2580 (82.8)	535 (65.9)	292 (65.9)	22,551 (88.4)	3209 (87.1)	341 (81.4)	191 (79.9)
Other	3080 (13.5)	537 (17.2)	277 (34.1)	151 (34.1)	2959 (11.6)	475 (12.9)	78 (18.6)	48 (20.1)
Education, Academic	11,164 (48.8)	1045 (33.5)	287 (35.3)	120 (27.1)	12,376 (48.5)	1082 (29.4)	139 (33.2)	54 (22.6)
Upper secondary/gymnasium	4867 (21.3)	639 (20.5)	172 (21.2)	78 (17.6)	5570 (21.8)	778 (21.1)	71 (16.9)	40 (16.7)
Lower secondary	4195 (18.3)	942 (30.2)	201 (24.8)	147 (33.2)	5306 (20.8)	1268 (34.4)	130 (31.0)	77 (32.2)
Primary	2660 (11.6)	491 (15.8)	152 (18.7)	98 (22.1)	2241 (8.8)	554 (15.0)	78 (18.6)	67 (28.0)
SEI^b^, Unskilled Workers	3223 (14.02)	586 (18.88)	170 (21.30)	99 (22.97)	3934 (17.35)	744 (22.84)	92 (25.14)	44 (21.15)
Skilled Workers	2141 (9.31)	399 (12.86)	108 (13.53)	68 (15.78)	3055 (13.48)	602 (18.48)	37 (10.11)	36 (17.31)
Lower non-manual	3058 (13.30)	454 (14.63)	111 (13.91)	58 (13.46)	2499 (11.02)	296 (9.09)	46 (12.57)	23 (11.06)
Intermediate non-manual	5829 (25.35)	738 (23.78)	155 (19.42)	79 (18.33)	4109 (18.13)	456 (14.00)	46 (12.57)	27 (12.98)
Higher non-manual	5394 (23.46)	503 (16.21)	116 (14.54)	46 (10.67)	3098 (13.67)	267 (8.20)	36 (9.84)	13 (6.25)
Self-employed	1738 (7.56)	275 (8.86)	59 (7.39)	37 (8.58)	1229 (5.42)	168 (5.16)	16 (4.37)	9 (4.33)
Other	1613 (7.01)	148 (4.77)	79 (9.90)	44 (10.21)	4746 (20.94)	725 (22.25)	93 (25.41)	56 (26.92)
Income, 1st (lowest) quintile	3905 (16.98)	403 (12.99)	233 (29.20)	155 (35.96)	5102 (22.51)	559 (17.16)	108 (29.51)	59 (28.37)
2nd	4858 (21.13)	572 (18.43)	228 (28.57)	101 (23.43)	487 (21.48)	619 (19.00)	103 (28.14)	53 (25.48)
3rd	4765 (20.72)	777 (25.04)	150 (18.80)	73 (16.94)	4612 (20.34)	643 (19.74)	68 (18.58)	35 (16.83)
4th	4761 (20.70)	724 (23.33)	108 (13.53)	56 (12.99)	4142 (18.27)	735 (22.56)	46 (12.57)	33 (15.87)
5th (highest)	4707 (20.47)	627 (20.21)	79 (9.90)	46 (10.67)	3944 (17.40)	702 (21.55)	41 (11.20)	28 (13.46)
Short-term unemployment before baseline (< 180 days), Yes	1887 (8.2)	253 (8.1)	110 (13.5)	67 (15.1)	3546 (13.9)	502 (13.6)	66 (15.8)	35 (14.6)
Unadjusted unemployment rates (cases)								
Any unemployment	33.6 (3819)	39.3 (572)	70.1 (225)	62.4 (102)	47.9 (6366)	55.6 (934)	70.7 (117)	79.2 (69)
≥90 days during 2 years	19.7 (2361)	24.5 (374)	42.7 (147)	36.6 (63)	29.6 (4384)	34.9 (646)	42.7 (81)	54.3 (52)
Person-years	113,516	14,543	3211	1634	132,876	16,797	1655	871

Categorical variables are frequencies and column proportions (%), and continuous variables are mean (SD) values. ^a^ The sum does not add up to the total number of participants since the overweight groups are not shown (full table available upon request to the corresponding author). ^b^ Swedish socio-economic index classification

### Statistical analysis

#### Any unemployment or long-term unemployment

A discrete-time proportional hazards model was used (information on unemployment was recorded annually) to examine differences in unemployment hazard ratios (HRs) with 95% confidence intervals (95% CIs) between study groups and normal weight people without MD (the reference group). Three different regression models with adjustment for different covariates were used: model 1 included adjustments for sex and age; model 2 had further adjustments for country of birth, educational level, and occupational status (main model); and model 3 had further adjustments for short-term unemployment before baseline (< 180 days). Models were stratified by survey period: 1996–2002, 2003–2007, and 2008–2012 in the ULF/SILC cohort, and 2002 and 2006 in the SPHS cohort. This stratification allowed the group-specific baseline hazards to vary between periods, and the cut-offs were chosen in order to account for possible macro-economic influences (such as the financial crisis in 2009) on unemployment hazards. Log-log plots and Schoenfeld residuals were used to assess the proportional hazards assumption. Additionally, from the proportional hazards models, post-estimation comparisons within cohorts and between groups other than the reference group were performed using the fully adjusted hazard models (Model 2).

#### Median unemployment (days on average)

Due to the skewed distribution of unemployment days, we used quantile regression to estimate median (95% CIs) unemployment days per year during follow-up, Poisson regression with robust variance was used to estimate relative risks (RR) and 95% CIs for being, on average, unemployed more than 30 days per year.

#### Group comparisons

We used the Wald test to test for differences between the group with MD *and* obesity (double exposure) and the groups with obesity only (single exposure) or MD only (single exposure). This comparison was done to explore whether MD or obesity was the strongest predictor in the associations under study. All analyses were carried out with STATA 14.1 (Stata Corp, College Station, TX).

## Results

### Baseline characteristics

The reported baseline results are based on all six exposure groups; however, the overweight groups are excluded in Table 1 to improve the readability. In the SPHS analytical sample (*n* = 40,088), 8.9% were obese (Table 1), and 5.0% had MD (Table 1). Almost 22% of the individuals in the groups with MD were obese compared with 8.1% in the groups without MD (Table 1). In the ULF/SILC analytical sample (*n* = 39,947), 8.8% were obese, and 2.5% had MD. Approximately 21% of the participants in the groups with MD were obese compared with 8.4% in the groups without MD. In both samples, individuals with MD more often were women, were older, had lower education, were employed in unskilled and non-manual type of labour, and had lower disposable income compared with members of the groups without MD (Table 1). The percentage of people born outside Sweden among the groups with MD was notably higher in the SPHS cohort.

### Any and long-term unemployment

In both cohorts, individuals of normal weight without MD had the lowest unemployment rate, and considerably higher rates were seen in the other groups, especially those with MD (Table 1).

During the follow-up period for the SPHS cohort (mean: 4.9 years; SD 2.2 years), 6676 (16.7%) individuals were unemployed at least once, whereas 4183 (10%) individuals were long-term unemployed. After adjusting for sex, age, country of birth, educational level, occupational status and income (Model 2, the main model), the results show that the obese group without MD and all the groups with MD had a higher relative risk of being unemployed (overall HR range: 1.30–1.59; 95% CI range: 1.06–1.90) compared with the reference group (Table 2). Short-term unemployment before baseline (< 180 days) was inversely related to subsequent unemployment (data not shown). After adjustments for short-term unemployment before baseline (Model 3) the overall trend in unemployment risks was similar to those observed in the main model (Model 2).

**Table 2:** Unemployment risk as any unemployment (upper part) and long-term unemployment (lower part) during the follow-up by mobility and weight status

	SPHS cohort, n = 40,088			ULF/SILC cohort, n = 39,947		
	Model 1, HRs (95% CIs)	Model 2, HRs (95% CIs)	Model 3, HRs (95% CIs)	Model 1, HRs (95% CIs)	Model 2, HRs (95% CIs)	Model 3, HRs (95% CIs)
Any unemployment						
No mobility disability						
Normal weight	Reference group	Reference group	Reference group	Reference group	Reference group	Reference group
Overweight	1.07 (1.00–1.13)	1.07 (1.01–1.14)	1.06 (1.00–1.13)	1.06 (1.01–1.11)	1.07 (1.03–1.13)	1.07 (1.02–1.12)
Obese	1.36 (1.24–1.49)	1.32 (1.20–1.45)	1.32 (1.20–1.45)	1.32 (1.23–1.42)	1.25 (1.15–1.34)	1.24 (1.15–1.34)
Mobility disability						
Normal weight	2.26 (1.96–2.61)	1.53 (1.33–1.77)	1.54 (1.32–1.78)	1.73 (1.42–2.10)	1.19 (0.97–1.45)	1.24 (1.01–1.53)
Overweight	2.19 (1.88–2.56)	1.35 (1.15–1.59)	1.39 (1.18–1.64)	1.69 (1.39–2.05)	1.17 (0.96–1.43)	1.22 (1.00–1.50)
Obese	2.33 (1.90–2.86)	1.40 (1.13–1.73)	1.44 (1.16–1.79)	2.00 (1.55–2.58)	1.26 (0.97–1.64)	1.40 (1.07–1.84)
Other group comparisons						
Test Nwt/MD vs. Ob/MD^1^	*p* = 0.82	*p* = 0.47	*p* = 0.61	*p* = 0.37	*p* = 0.72	*p* = 0.48
Test Ob/noMD vs. Ob/MD^2^	*p* < 0.001	*p* = 0.61	*p* = 0.47	*p* = 0.002	*p* = 0.94	*p* = 0.40
Long-term unemployment						
No mobility disability						
Normal weight	Reference group	Reference group	Reference group	Reference group	Reference group	Reference group
Overweight	1.04 (0.97–1.12)	1.04 (0.97–1.12)	1.04 (0.96–1.12)	1.09 (1.04–1.16)	1.11 (1.05–1.17)	1.09 (1.03–1.16)
Obese	1.38 (1.23–1.54)	1.33 (1.19–1.49)	1.33 (1.19–1.49)	1.30 (1.19–1.42)	1.22 (1.12–1.34)	1.20 (1.10–1.32)
Mobility disability						
Normal weight	2.37 (1.99–2.82)	1.59 (1.33–1.90)	1.58 (1.32–1.90)	1.68 (1.33–2.12)	1.15 (0.91–1.46)	1.18 (0.93–1.51)
Overweight	2.08 (1.71–2.53)	1.30 (1.06–1.59)	1.34 (1.09–1.65)	1.62 (1.29–2.03)	1.11 (0.88–1.41)	1.18 (0.93–1.50)
Obese	2.27 (1.75–2.94)	1.40 (1.07–1.83)	1.37 (1.05–1.80)	2.16 (1.61–2.89)	1.34 (0.99–1.81)	1.50 (1.11–2.05)
Other group comparisons						
Test Nwt/MD vs. Ob/MD	*p* = 0.16	*p* = 0.41	*p* = 0.38	*p* = 0.17	*p* = 0.43	*p* = 0.22
Test Ob/noMD vs. Ob/MD	p < 0.001	*p* = 0.73	*p* = 0.83	*p* = 0.001	*p* = 0.55	p = 0.17

Values are hazard ratios (HRs) and 95% confidence interval (95% CIs) of any unemployment (≥1 day) and long-term unemployment (≥90 days during two consecutive years) estimated by a stratified discrete proportional hazards model compared to normal weight without MD (the reference group). Individuals who were unemployed for 180 days or more before the start of follow-up or who received any disability pension were excluded. Individuals who received any disability pension, who died who emigrated who turned 65 years or who did not become unemployed before the end of follow up (2012) were censored

Model 1: adjusted for sex and age (demographic factors)

Model 2: + country of birth, educational level, occupational status, and income (socio-economic factors)

Model 3: + short-term unemployment at baseline (< 180 days)

1. Testing differences between the group with MD and obesity (double exposure) and the group with MD only (single exposure)

2. Testing differences between the group with MD and obesity (double exposure) and the group with obesity only (single exposure)

During the follow-up period for the ULF/SILC cohort (mean: 5.7 years; SD: 4.2 years), 10,845 (27%) individuals were unemployed at least once, whereas 7590 (19%) individuals were long-term unemployed. Results from Model 2 (the main model) show the obese group without MD had a higher relative risk of being unemployed compared with the reference group (Table 2). Similar relative risks were observed for the groups with MD (HR range: 1.11–1.34; 95% CI range: 0.88–1.81) compared with the reference group. Short-term unemployment before baseline (< 180 days) was, as in the SPHS cohort, inversely related to subsequent unemployment (data not shown). After adjustments for short-term unemployment before baseline (Model 3) similar results to those of the main model (Model 2) were observed, although the relative risk appeared to increase for the obese group with MD. Notably the obese group without MD had a stable relative risk increase (20–30%) of being unemployed short- and long-term compared with the reference group regardless of adjustments made.

#### Results from group comparisons for both the SPHS and ULF/SILC cohorts

After adjustments for sex and age (Model 1) the results showed that obese group with MD had a statistically significant higher relative risk (based on Wald test with associated *p* > 0.05) compared with the obese group without MD. However, after further adjustments for country of birth, educational level, occupational status and income, no statistically significant relative risk differences remained between the obese group with MD and the group with obesity only or MD only.

### Days of unemployment

For the SPHS cohort results (calculated from Table 3, upper part) show, after adjustments for sex, age, country of birth, educational level, occupational status and income (Model 2, the main model), that the two obese groups had, on average, more unemployment days per year than the normal weight group without MD (reference group). The obese group with MD had notably more unemployment days on average (median: 13.8; 95% CI: 4.4–23.2) than the reference group. Further, the results from the main model (Table 3, lower part) show that the obese group without MD and the normal weight group with MD had an approximately 20% higher relative risk (RR range: 1.24–1.25; 95% CI range: 1.07–1.46) of being unemployed more than 30 days than the reference group. After adjustments for short-term unemployment before baseline (< 180 days, Model 3) the overall trend in unemployment risks was similar to those observed in the main model (Model 2).

**Table 3:** Unemployment risk as average accumulated unemployment days during follow-up by mobility and weight status

	SPHS cohort, n = 40,088			ULF/SILC cohort, n = 39,947		
Days per year on average	Model 1, Median (95% CIs)	Model 2, Median (95% CIs)	Model 3, Median (95% CIs)	Model 1, Median (95% CIs)	Model 2, Median (95% CIs)	Model 3, Median (95% CIs)
No mobility disability						
Normal weight	31.9 (30.5–33.2)	33.3 (31.9–34.6)	34.6 (33.2–36.0)	26.4 (25.3–27.4)	27.6 (26.5–28.7)	28.5 (27.4–29.5)
Overweight	32.9 (30.9–34.9)	33.8 (31.8–35.8)	35.4 (33.3–37.5)	27.4 (25.9–28.9)	28.6 (27.1–30.1)	29.4 (28.0–30.9)
Obese	37.4 (33.8–41.0)	38.3 (34.7–41.9)	41.4 (37.7–45.1)	30.0 (27.3–32.7)	29.2 (26.4–32.0)	30.0 (27.3–32.7)
Mobility disability						
Normal weight	42.4 (36.4–48.4)	37.1 (31.1–43.2)	38.6 (32.3–44.9)	30.3 (22.6–37.9)	25.5 (17.6–33.5)	29.2 (21.6–36.7)
Overweight	41.6 (35.1–48.2)	38.0 (31.4–44.6)	35.4 (28.5–42.2)	26.4 (18.8–33.9)	26.1 (18.2–33.9)	27.0 (19.5–34.5)
Obese	54.9 (45.6–64.2)	47.0 (37.7–56.4)	48.5 (38.8–58.2)	38.2 (28.2–48.1)	35.7 (25.3–46.0)	37.5 (27.7–47.4)
30 days or more per year on average	Model 1, RRs (95% CIs)	Model 2, RRs (95% CIs)	Model 3, RRs (95% CIs)	Model 1, RRs (95% CIs)	Model 2, RRs (95% CIs)	Model 3, RRs (95% CIs)
No mobility disability						
Normal weight	Reference group	Reference group	Reference group	Reference group	Reference group	Reference group
Overweight	1.06 (0.99–1.13)	1.05 (0.98–1.12)	1.04 (0.98–1.11)	1.08 (1.01–1.15)	1.07 (1.00–1.14)	1.05 (0.98–1.12)
Obese	1.31 (1.18–1.44)	1.24 (1.12–1.37)	1.23 (1.11–1.35)	1.29 (1.17–1.43)	1.19 (1.08–1.32)	1.18 (1.07–1.30)
Mobility disability						
Normal weight	1.86 (1.59–2.17)	1.25 (1.07–1.46)	1.26 (1.07–1.47)	1.56 (1.21–2.02)	1.07 (0.83–1.39)	1.07 (0.82–1.38)
Overweight	1.85 (1.58–2.18)	1.14 (0.97–1.35)	1.15 (0.98–1.36)	1.49 (1.16–1.93)	1.02 (0.79–1.32)	1.07 (0.83–1.38)
Obese	1.80 (1.44–2.24)	1.09 (0.87–1.36)	1.06 (0.85–1.33)	2.04 (1.51–2.76)	1.31 (0.97–1.77)	1.38 (1.02–1.87)

Upper part: Results from quantile regression showing the average number of unemployment days per year during follow-up, represented as group median values (95% CIs). Lower part: Results from Poisson regression with robust standard errors showing the relative risks (95% CIs) of having 30 unemployment days or more per year on average compared to normal weight without MD (the reference group). Individuals who were unemployed for 180 days or more before baseline or who received any disability pension were excluded

Model 1: adjusted for sex and age (demographic factors)

Model 2: + country of birth, educational level, occupational status, and income (socio-economic factors)

Model 3: + short-term unemployment at baseline (< 180 days)

For the ULF/SILC cohort results from the main model (Model 2; Table 3, upper part) show no statistically significant differences in average (median) unemployment days between any of the exposure groups and the reference group. There was; however, an indication of more unemployment days on average for the obese group with MD than for the reference group (Table 3, upper part). Further, the results from the main model (Table 3, lower part) showed a higher relative risk of being unemployed more than 30 days for the obese non-disabled group compared with the reference group (RR = 1.19; 95% CI = 1.08–1.32). A similar result was observed between the obese group with MD and the reference group (RR = 1.31; 95% CI = 0.97–1.77). Adjusting further for short-term unemployment before baseline (< 180 days, Model 3) did not materially change the estimates observed from the main model (Table 2).

## Discussion

### Main findings

This study followed two population-based cohorts over a long period of time which included vulnerable groups of people with obesity and MD at risk of unemployment. Two different definitions of MD were used and several unemployment risk outcomes were investigated. We observed an overall trend of increased unemployment risk for all the exposure groups compared with normal weight people without MD. The results were similar and robust for both cohorts. Further, no excess unemployment risk of MD and obesity combined was observed beyond the excess unemployment risk of MD or obesity alone. Instead, MD and obesity remained separate risk factors after taking into account healthy worker selection into work as well as confounding by various socio-demographic factors.

### No apparent excess burden of co-existing MD and obesity in terms of unemployment risk

People with MD *and* obesity (double exposed group) were not at higher risk of unemployment than people with MD only or obesity only (single exposed groups). The assumption was that over time people with MD and obesity risk more severe comorbidities, and perhaps other social adversities, that would put them at higher risk of being unemployed compared to having only one of the conditions. An association of poor health and subsequent unemployment has been demonstrated. A study of Finnish middle-aged men with lower occupational status (construction workers) has demonstrated that both various health problems (OR range: 1.97–7.75; 95% CI range: 1.01–39.93) and employment history (OR: 2.13; 95% CI: 1.20–3.80) independently predicted long-term unemployment [35]. Another study found that middle-age people with poor self-rated health, especially with physical disability such as musculoskeletal pain (OR: 1.93; 95% CI: 1.65–2.27), were at an exceptionally great risk for long-term unemployment [36]. We tried to account for potential comorbidities between the double- and single-exposed groups by excluding people with long-term unemployment before follow-up started. Moreover, in Sweden disability benefits are, since 2003, almost exclusively awarded to individuals with permanently reduced work capacity associated with a medical diagnosis. By also excluding people with any form of disability benefits (most often people with MD) this would, at best, help to ensure that any potential long-term unemployment differences between the double- and single-exposed groups were not due to a history of poor health. Thus, in light of the study findings, it is possible that any unemployment differences between the double- and single-exposed groups are best explained by group differences in socio-demographic factors and comorbidities.

Further, people living in Sweden who have longer or permanent employment contracts are protected against job termination due to poor health, but it is possible that potential differences in health between the double- and single-exposed groups could influence unemployment risk for individuals on shorter and temporary work contracts. Unfortunately, more detailed information, such as the employment type (permanent or temporary) and length (full-time or part-time), was unavailable; thus, we can only speculate whether potential health differences between the double- and single-exposed groups could influence subsequent unemployment risk differently depending on the job individuals hold. Further, qualitative information on reasons for being unemployed could also be valuable when investigating how conditions such as MD and obesity with poor health influence future unemployment risk between groups, but this was outside the objective of this quantitative study. Another possibility is that people with MD may experience more severe comorbidities with increasing obesity levels (BMI ≥ 35) that would put them at greater risk of being unemployed. The study samples had few respondents with more severe obesity levels (low statistical power), which could explain why we did not observe any noticeable relative risk difference between the double- and single-exposed groups. Last, it is possible that any excessive health problems potentially experienced by the double-exposed group, compared with the single-exposed groups, are more likely to increase their risk of being permanently excluded from the work force through disability benefits, rather than contributing to a greater risk of unemployment. Such relationships has been more frequently observed in the Nordic countries with highly developed welfare systems [13, 24].

### High unemployment rates of people with MD and/or obesity

We found that people with obesity, and people with MD regardless of weight status, are groups who are more likely to be unemployed than people without these conditions. The results were robust and of similar magnitude between the study cohorts despite being different in terms of the MD definition used. The results are in line with previous research investigating other aspects of work participation in similar groups [12, 26, 27, 37, 38]. One study found that people with MD had approximately 2–4 fold higher odds of not participating on the labour market than people without MD during 8 years of follow-up [12]. Another study showed that people with MD were much less likely to be working than those with no or other disabilities (OR: .28, 95% CI: .21–.38; *p* < .0001) [38]. The association of obesity as a risk factor for subsequent unemployment is poorly understood, and not without contradiction. While a study found a higher risk of unemployment in women (OR: 2.0, 95% CI: 1.2–3.4), but not in men [39], two other studies found no meaningful association of being obese and subsequent risk of unemployment, after taking into account multiple confounding factors [40, 41]. Further, work-related factors such as a work accommodations, including flexible working schedules, and supportive work environments, have been suggested to play a key role in retaining people with MD and/or obesity [26, 29, 32, 37]. Low occupational status has been associated with cumbersome and less-skilled occupations with poorer conditions and non-supportive work environments [42]. People with MD and/or obesity are more likely to hold these types of jobs [37, 43], partly due to lower education and financial problems, possibly because of increased health care expenditures. Although detailed information on work-related factors was unavailable in this study, information on occupational status (besides educational level) was used to account for some of the confounding effect of work-related factors, but it is likely that some unmeasured and residual confounding remains.

The potential confounding of short-term unemployment (defined as less than 180 unemployment days) before baseline, on the associations under study was explored [28, 33, 34]. The results show that short-term unemployment before baseline was higher in the groups with MD than in the groups without it, especially the SPHS cohort, as well as inversely associated to subsequent unemployment in both cohorts. The SPHS is a population-based cohort from the county of Stockholm. Since people with MD are more likely to hold temporary and insecure job contracts [28, 43] than people without MD, they may be more likely to be unemployed in an urbanized setting where the labour market concentrates and where job competition is high. Moreover, the influence of the economic downturn during the study period may have had a greater impact on unemployment for people MD [28]. Nevertheless, based on both the data available in this study and follow-up time, it is very hard to disentangle both the temporality and potential causal mechanisms of short-term unemployment before baseline on chronic conditions such as obesity and MD; thus, we can only speculate about its influence. Most importantly, the overall unemployment risk differences between the groups in both cohorts were of similar magnitude when comparing a model including short-term unemployment before baseline to a model without it (the main model of this paper). Moreover, the results indicate that adjusting for short-term unemployment before baseline may further increase the unemployment risk in the groups with MD (bias away from the null).

Another important aspect to consider is the type, severity and duration of the condition underlying a reported MD in the study participants. Apart from accidents and other traumas, there are many physical, mental, and other chronic health states that may underlie a disabling condition [44]. In a study from New Zealand, it was suggested that the longer people were disabled, the higher was their risk of being unemployed [45]. In a Korean study of people with MD, the probability of being unemployed increased dramatically with increasing MD severity [27]. Moreover, there is some evidence from working age populations showing that unemployment risk increases the longer people have lived with obesity, and this association appears stronger in women [46–48]. It is likely that these factors have a strong influence on the association between MD and obesity with the risk of unemployment over the life-course, although we were unable to investigate the magnitude and direction of such an influence in the current study.

Employer prejudice and discrimination may explain part of the unemployment gap observed in this study between people with MD and/or obesity and those without these conditions [29, 31]. However, it is difficult to separate the impact of discrimination from that of health-related productivity differences between the groups in this study. Prejudice and discrimination may act both on wage and on employment prospects for people with MD and/or obesity, but the impact varies by factors such as gender, and the severity and type of the disabling condition.

In addition to residual confounding, it is possible that other factors influenced the association investigated in this study. It is; however, beyond the scope of the current paper to summarize and discuss all potential pathways to unemployment for people with MD and/or obesity, which represents a task better left for systematic review.

### Strengths and limitations

The current study used two large population-based samples of the Swedish working population, which allowed for the identification of rather large groups of people with MD and/or obesity. Further, using different definitions of MD between the cohorts allowed investigation of the robustness of the findings. The study participants were followed up over a reasonably long period of time in a national register with high coverage and almost no loss to follow-up regarding objective unemployment outcomes [22] for people of working age who live in Sweden.

Some limitations need to be discussed. First, non-response is always a problem when using information from population-based surveys. In the ULF/SILC surveys, non-participation has increased in the last decade and reached levels beyond 50% in 2013 [20]. The SPHS surveys had participation rates of approximately 60% in 2002 and 2006. Non-response rates have been higher for people of younger ages, with less education, and who were born outside Sweden. In the current study, younger participants were more often found in the group without MD and with normal weight, whereas participants with lower education and those born outside of Sweden were over-represented in the groups with MD and/or overweight. Even after accounting for these confounding factors in the regression models, it is difficult to exclude residual confounding. Second, information about the underlying comorbidities of MD and obesity, such as cancer, CVD, and diabetes were not included. It is likely that these factors mediate the association of MD and obesity with risk of unemployment, but this lies outside the study objective and would therefore wrongfully attenuate the estimated association of interest. Moreover, these factors certainly influence more permanent pathways out of the workforce for people with MD and/or obesity, for instance through disability benefits [15]. Instead, we tried to account for bias of health selection into unemployment [35, 36] by excluding people who were on disability pension or who had been long-term unemployed for more than, or equal to, 180 days before the start of follow-up. Last, information on aspects of MD is often limited in health surveys, and thus its definition and prevalence differ between countries [1]. In this study, information on mobility status was obtained from self-reports, but with the possibility of using two rather different definitions of MD, which yielded similar results between the study cohorts. Information on BMI was also calculated using self-reported height and weight, and misreporting has been shown to exist with respect to weight and height in population-based surveys [49]. Further, more accurate information on body size and fat mass through measures of bioelectrical impedance and waist circumference could have provided a more accurate obesity prevalence among people with MD [15, 50]. In the current study, the prevalence of severe obesity is most likely underestimated, which may partly explain why we did not observe any clear differences in unemployment rates between the groups with MD.

## Conclusions

In this study, MD and obesity were found to be independently associated with an increased risk of unemployment; however, the co-existence of MD and obesity did not seem to further increase the unemployment risk. The results from two different cohorts with different definitions of MD and with geographical and demographic differences were found to be robust and consistent, which increases the generalizability of this study. The presented patterns are likely to be similar to those in other Scandinavian countries with comparable social welfare systems, work integrating measures, and work policies. Several potential explanations for the observed unemployment gap have been considered; however, more longitudinal studies are needed that use information on the type, severity and duration of interacting underlying conditions of MD and, preferably, repeated measures of weight and height, starting from young adulthood and continuing to late working age. These studies also must distinguish between the influence of poor health and discrimination on unemployment risks in these groups of people. This strategy may help to allocate public resources more effectively and tailor interventions carried out by the concerted effort of health workers and stake holders at all levels of society, ultimately improving the work participation of much-overlooked populations with MD and/or obesity.

## Additional files

Additional file 1: ULF_SILC data deidentified. (XLSX 11.7 mb)

Additional file 2: SPHS data deidentified. (XLSX 11.1 mb)

## Abbreviations

BMI: Body mass index
LISA: Longitudinal Integration Database for Health Insurance and Labour Market Studies
MD: Mobility disability
SPHS: Stockholm Public Health Surveys
ULF/SILC: National Survey of Living Conditions
